# Examining the effectiveness of zinc treatment in children admitted with diarrhoea in Kenya’s public hospitals: an observational comparative effectiveness study

**DOI:** 10.7189/jogh.09.020416

**Published:** 2019-12

**Authors:** Lucas Malla, Rafael Perera-Salazar, Samuel Akech, Morris Ogero, Thomas Julius, Grace Irimu, Mike English, on behalf of the CIN author group

**Affiliations:** 1 Nuffield Department of Medicine, University of Oxford, Oxford, UK; 2 Nuffield Department of Primary Care Health Sciences, University of Oxford, Oxford, UK; 3 Kenya Medical Research Institute-Wellcome Trust Research Programme, Nairobi, Kenya; * Listed in the Authorship contribution section

## Abstract

**Background:**

Kenyan paediatric treatment protocols recommend the use of zinc supplement for all children with diarrhoea. However, there is limited evidence of benefit for young children aged 1-5 months and those who are well-nourished. We examine effectiveness of zinc supplementation for children admitted with diarrhoea to Kenya’s public hospitals with different nutritional and age categories. This is to determine whether the current policy where zinc is prescribed for all children with diarrhoea is appropriate.

**Methods:**

We explore the effect of zinc treatment on time to discharge for children aged 1-5 and 6-59 months and amongst those classified as either severely – moderately under-nourished or well-nourished. To overcome the challenges associated with non-random allocation of treatments and missing data in these observational data, we use propensity score methods and multiple imputation to minimize bias.

**Results:**

The analysis included 1645 (1-5 months) and 11 546 (6-59 months) children respectively. The estimated sub-distribution hazard ratios for being discharged in the zinc group vs the non-zinc group were 1.25 (95% confidence interval (CI) = 1.07, 1.46) and 1.17 (95% CI = 1.10, 1.24) in these respective age categories. Zinc treatment was associated with shorter time to discharge in both well and under-nourished children.

**Conclusion:**

Zinc treatment, in general, was associated with shorter time to discharge. In the absence of significant adverse effects, these data support the continued use of zinc for admissions with diarrhoea including those aged 1-5 months and in those who are well-nourished.

Diarrhoea is a major cause of morbidity and mortality in lower and middle income countries with the primary treatment being rehydration regimens matched to the severity of the dehydration [1,2]. Co-treatment with zinc supplements is also recommended [2,3]. This is based on results of randomised controlled trials and subsequent systematic reviews [4-8] suggesting that zinc supplements reduce diarrhoea duration for children aged six months and above. Kenyan guidelines in keeping with those of the World Health Organisation [WHO] recommend treatment of diarrhoea with fluids and co-treatment with oral zinc for 14 days for all children irrespective of illness severity [2].

Despite these recommendations, there remains some debate on the benefits of zinc since:

- The prevalence of zinc deficiency varies by settings [9] and this may contribute to between country variations in zinc effectiveness reported in a systematic review by Patel (2012) [10].
- Trials supporting the use of zinc have included fewer participants from Africa than other low and middle income settings [7] and it is suggested that African children with diarrhoea have poor health outcomes [1].
- There are few data supporting the benefits of zinc in children younger than six months and few data on the effects of zinc in children of all ages who are relatively well-nourished [7].

Conducting trials to address all these questions would likely be expensive and time-consuming. Appropriate analyses of observational data sets may help address such questions while also providing data on the effectiveness of zinc treatment in non-trial populations.

## Objectives

The primary focus of this analysis is to examine the effectiveness of zinc supplementation, assuming those prescribed zinc actually received it, in reducing time to discharge for children admitted with diarrhoea in Kenyan hospitals. In secondary analyses, we aim to examine the effectiveness of zinc amongst those classified as either severely-moderately under-nourished or well-nourished.

## METHODS

### Study design and data source

We use observational data from the Kenyan Clinical Information Network (CIN) that was initiated in September 2013 to improve inpatient paediatric data availability from 14 hospitals. These hospitals typically have one paediatrician leading services predominantly provided by junior clinical teams. Data in these hospitals are collected post discharge by trained data clerks guided by well-defined standard operating procedures, under supervision by the hospital medical records department and the research team. Clinicians admitting patients fill standardized Paediatric Admission Record (PAR) forms [11] that have been shown to improve documentation of clinical symptoms and signs [12]. Together with discharge forms, treatment sheets and laboratory reports these are all part of the patient files that are the primary data source. Information on zinc prescription was abstracted from the treatment sheets when clinicians indicated to have prescribed this treatment. The data used for this analysis are those collected between October 2013 and February 2017. More information on CIN is provided in the supplementary material.

### Statistical analysis

#### Analysis populations

We include in the analysis all children who had diarrhoea as one of the illnesses on admission. We exclude children aged <1 month and ≥60 months, had shock or who had impaired consciousness (response only to pain or unresponsive), and those who had a clinical diagnosis of severe acute malnutrition. We primarily examine the effect on time to discharge of whether children were prescribed zinc in two age groups: all children aged 1-5 months (group 1) and all children aged 6-59 months (group 2). The analysis is stratified into two age groups as these are the age groups considered in the guidelines, with different zinc prescription recommendations. Within each of these groups, we examine the effects of zinc treatment in severely-moderately under-nourished and well-nourished children. To define nutritional status in this population not clinically identified as having severe acute malnutrition, we derive weight for age z scores using WHO reference population data [13]. Children with weight for age z-scores<-2 are classified retrospectively as severely-moderately under-nourished, while those with z-scores≥-2 are considered well-nourished [13].

#### Handling of missing data

As there were missing data in the variables (listed in **Table 1**) we use for analysis (see Table S1 in **Online Supplementary Document** for data completeness (%)), we assume these data were missing at random (MAR) and to maintain the effective sample size, multiple imputation methods are used to fill in the missing data [14]. In this approach, we impute 10 data sets [15] using chained equations which allow for the correct specification of the distribution of each variable. We then examine the plausibility of imputed values by comparing the distribution of observed values with that of imputed values (see Figure S1 in **Online Supplementary Document**).

**Table 1:** Distribution of patients by clinical signs, co-treatments, co-morbidities and hospital (before and after propensity score weighting)

	Group 1 (1-5 months)						Group 2 (6-59 months)					
**Variable**	**Before PS weighting**			**After PS weighting**			**Before PS weighting**			**After PS weighting**		
	**Zinc (n = 1181)**	**No zinc (n = 464)**	**ASMD**	**Zinc**	**No zinc**	**ASMD**	**Zinc (n = 8853)**	**No zinc (n = 2693)**	**ASMD**	**Zinc**	**No zinc**	**ASMD**
**Discrete variables**												
**Pulse:**			0.04			0.06			0.08			0.03
Normal	93.9	93.7		93.9	92.9		94.6	92.7		94.6	93.8	
Weak	6.1	6.3		6.1	7.1		5.4	7.3		5.4	6.2	
**AVPU:**			0.07			0.09			0.06			0.00
Alert	97.8	96.6		97.8	97.4		97.7	97.0		97.7	97.7	
Verbal response	2.2	3.4		2.2	2.6		2.3	3.0		2.3	2.3	
**Capillary refill:**			0.12			0.03			0.03			0.00
≤3 seconds	95.6	96.3		95.6	95.0		95.3	94.4		95.3	95.3	
>3 seconds	0.7	1.5		0.7	0.8		1.1	1.4		1.1	1.1	
Intermediate	3.7	2.2		3.7	4.2		3.6	4.2		3.6	3.6	
**Sunken eyes:**			0.18			0.06			0.15			0.01
Yes	29.0	20.6		29.0	26.3		33.6	26.8		33.6	32.6	
No	71.0	79.4		71.0	73.7		66.4	73.2		66.4	67.4	
**Skin pinch:**			0.03			0.07			0.05			0.02
1–2 seconds	23.0	22.4		23.0	20.1		22.9	21.4		22.9	22.6	
2> seconds	67.7	68.0		67.7	72.1		8.1	9.2		8.1	8.6	
Immediate	9.3	9.6		9.3	7.8		69.0	69.4		69.0	68.8	
**Blood transfusion order:**			0.01			0.05			0.10			0.02
Yes	2.9	2.8		2.9	2.5		2.4	4.0		2.4	2.7	
No	97.1	97.2		97.1	97.5		97.6	96.0		97.6	97.3	
**Able to drink:**			0.14			0.06			0.06			0.00
Yes	84.4	78.7		84.4	82.2		84.7	82.6		84.7	84.7	
No	15.6	21.3		15.6	17.8		15.3	17.4		15.3	15.3	
**Skin temperature:**			0.11			0.06			0.05			0.02
Elbow	3.0	4.1		3.0	4.0		3.6	4.0		3.6	3.8	
Hand	95.4	92.9		95.4	94.5		94.3	93.4		94.2	94.0	
Shoulder	1.6	3.0		1.6	1.5		2.1	2.6		2.2	2.2	
**Child sex:**			0.01			0.03			0.05			0.01
Male	55.2	54.6		55.2	54.3		55.2	53.1		55.2	55.8	
Female	44.8	45.4		44.8	45.7		44.8	46.9		44.8	44.2	
**Pallor:**			0.12			0.06			0.14			0.02
None	87.8	85.8		87.8	86.9		88.0	83.4		88.0	87.6	
Mild/moderate	9.2	12.3		9.2	9.9		9.5	12.3		9.5	9.8	
Severe	3.0	1.9		3.0	3.1		2.5	4.3		2.5	2.7	
**Fever:**			0.07			0.07			0.01			0.05
Yes	81.3	78.7		81.3	78.7		75.5	75.9		75.5	11.4	
No	18.7	20.3		18.7	20.3		24.5	24.1		24.5	89.6	
**Convulsions:**			0.05			0.04			0.08			0.01
Yes	7.5	8.6		7.5	7.9		11.2	13.2		11.2	11.4	
No	92.5	91.4		92.5	92.1		88.8	86.8		88.8	88.6	
**Vomiting:**			0.05			0.10			0.17			0.01
Yes	60.6	58.1		60.6	55.6		81.3	74.7		81.3	80.9	
No	39.4	41.9		39.4	44.4		18.7	25.3		18.7	19.1	
**Hospital referral:**			0.24			0.01			0.17			0.01
Yes	17.2	27.1		17.2	17.0		16.4	21.8		16.4	16.9	
No	82.8	72.9		82.8	83.0		83.6	78.2		83.6	83.1	
**Severe wasting:**			0.19			0.04			0.18			0.01
Yes	5.6	11.2		5.6	5.2		5.7	10.4		5.7	5.9	
No	94.4	88.8		94.4	94.8		94.3	89.6		94.3	94.1	
**Thrush:**			0.03			0.01			0.08			0.01
Yes	3.9	4.3		3.9	4.0		3.4	4.9		3.4	3.1	
No	96.1	95.7		96.1	96.0		96.6	95.1		96.6	96.9	
**Oedema:**			0.09			0.10			0.05			0.01
Face	0.2	0.0		0.2	0.0		0.3	0.4		0.3	0.4	
Foot	1.1	1.0		1.1	1.5		1.3	1.8		1.3	1.4	
Knee	0.2	0.2		0.2	0.2		0.1	0.2		0.1	0.1	
None	98.5	98.8		98.5	98.3		98.3	97.6		98.3	98.1	
**Oral fluid:**			0.26			0.03			0.32			0.00
Administered	83.8	75.1		83.8	83.7		87.9	75.2		87.9	88.0	
Not administered	16.2	24.9		16.2	16.3		12.1	24.8		12.1	12.0	
**IV fluid:**			0.02			0.07			0.07			0.00
Administered	35.5	36.8		35.5	37.4		33.7	37.9		33.7	33.8	
Not administered	64.5	63.2		64.5	62.6		66.3	62.1		66.3	66.2	
**Wheeze:**			0.16			0.01			0.10			0.00
Present	3.7	7.8		3.7	3.6		2.1	3.4		2.1	2.1	
Absent	96.3	92.2		96.3	96.4		97.9	96.6		97.9	97.9	
**Hospital:**			0.74			0.13			0.59			0.05
H 1	10.2	2.4		10.2	7.4		7.9	4.1		7.9	7.5	
H 2	8.6	8.6		8.6	8.3		9.2	9.1		9.2	9.4	
H 3	10.8	8.0		10.8	12.2		9.5	10.8		9.5	9.3	
H 4	5.3	4.5		5.3	6.1		6.9	6.7		6.9	6.7	
H 5	4.8	3.9		4.8	4.2		5.6	5.8		5.6	5.8	
H 6	11.9	5.2		11.9	11.3		12.1	6.8		12.1	11.7	
H 7	6.5	4.3		6.5	6.2		7.1	5.4		7.1	7.2	
H 8	11.9	9.7		11.9	12.3		14.6	9.5		14.6	15.6	
H 9	7.3	7.5		7.3	7.7		6.0	4.2		6.0	5.7	
H 10	11.3	15.3		11.3	12.1		7.7	9.6		7.7	7.9	
H 11	3.7	20.5		3.7	3.8		2.6	15.6		2.6	2.7	
H 12	0.5	0.0		0.5	4.6		0.5	1.3		0.5	0.5	
H 13	3.9	8.6		3.9	2.6		5.9	8.4		5.9	6.0	
H 14	3.3	1.4		3.3	1.2		4.4	2.7		4.4	4.0	
**Diarrhoea >14 days:**			0.03			0.02			0.04			0.01
Yes	3.0	1.9		3.0	2.6		2.9	3.9		2.9	2.7	
No	97.0	98.1		97.0	97.4		97.1	96.1		97.1	97.3	
**HIV:**			0.07			0.02			0.06			0.01
Positive	1.3	2.2		1.3	1.3		1.4	2.4		1.4	1.5	
Negative	98.7	97.8		98.7	98.7		98.6	97.6		98.6	98.5	
**Pneumonia:**			0.19			0.01			0.19			0.02
Positive	49.4	58.7		49.4	49.8		28.4	36.1		28.4	27.6	
Negative	50.6	41.3		50.6	50.2		71.6	63.9		71.6	72.4	
**Malaria:**			0.22			0.00			0.09			0.01
Positive	23.1	13.5		23.1	23.0		29.0	24.9		29.0	28.7	
Negative	76.9	86.5		76.9	77.0		71.0	75.1		71.0	71.3	
**Meningitis:**			0.18			0.01			0.09			0.01
Positive	4.1	8.4		4.1	4.5		4.7	6.8		4.7	4.8	
Negative	95.9	91.6		95.9	95.5		95.3	93.2		95.3	95.2	
**Continuous variables**												
Weight – kg (mean)	5.8	5.6	0.13	5.8	5.8	0.00	10.2	10.8	0.04	10.2	10.2	0.01
Temperature (mean)	37.7	37.9	0.20	37.7	37.7	0.02	37.7	37.7	0.06	37.7	37.7	0.01
Length of illness – days (mean)	5.4	5.2	0.01	5.4	5.4	0.05	4.8	5.9	0.10	4.8	4.9	0.01

PS – propensity score, ASMD – absolute standardised mean difference, AVPU – alert, verbal, pain, unresponsive

*Summary of discrete variables are presented as percentages while continuous variables are presented as means.

The plausibility of MAR assumption is examined by conducting sensitivity analyses under missingness not at random (MNAR) assumption [16]. Under MAR, we assume the clinicians unintentionally failed to record such clinical data or fill some sections of the treatment sheets and the missingness process is therefore ignorable thus the recorded data by clinicians would plausibly be predictive of missing data [16]. However, it is also possible that data are missing for reasons that are not random but linked to the data recording process. These reasons are not necessarily known but could create a scenario in which data are MNAR. See the supplementary material for the MNAR patterns considered.

#### Handling non-random allocation of zinc supplement

As CIN comprises data from routine care settings, not being prescribed zinc cannot be considered random. In fact, this would be as a result of failure to adhere to national guidelines. Analyses must try and account for any potential bias in treatment allocation. To help create balance in population characteristics amongst those with and without zinc prescription, we compare propensity score full-matching and weighting [17,18]. A propensity score describes the probability of a patient being prescribed zinc based on measured characteristics, in this case clinical signs, symptoms, co-treatments and comorbidities [18]. Full-matching and weighting adjustments are done for each of the 10 imputed data sets and absolute standardised mean differences (ASMD) used as diagnostic checks for covariate balance and overlap [19,20] between zinc and non-zinc groups. We then base outcome analysis on the best performing method resulting in minimised ASMD for most of the variables. Covariables used in creating propensity scores in all the analyses include key signs and symptoms suggested for diagnosing and assessing severity of diarrhoea and dehydration in the Kenyan paediatric guidelines together with variables considered *a priori* to influence the clinical outcomes of interest such as fluid regimen prescribed and comorbidities (see Table S1 in **Online Supplementary Document** for a summary of these variables, and *sub section (d)* of **Online Supplementary Document** for the process of fitting the propensity score models).

#### Modelling of time to being discharged alive

We consider time to being discharged alive as the primary outcome. Mortality is treated as a competing risk as it would preclude the chance of a patient being discharged alive. To allow for covariates with varying effects across the discharge time points, we use a flexible modelling approach suggested by Scheike (2011) [21]. Both Kolmogorov-Smirnov and Cramer von Mises test statistics [21] are used to determine whether covariates have varying or constant effects across time points prior to discharge. Patients who absconded, were referred to other hospitals and those who had length of stay greater than 15 days (as zinc should be administered for two weeks and should influence the outcome of acute diarrhoea) are censored.

For each of the imputed and then propensity score adjusted data sets, Scheike’s flexible regression models are fitted while adjusting for all the variables (see Table S1 in **Online Supplementary Document**) also used in the corresponding propensity score models. Only adjusted treatment effectiveness estimates are pooled across the imputed data sets using Rubin rules [22] to obtain a single estimate. This analysis also reports estimates without propensity score adjustments. An additive interaction [23] is used in modelling the use of zinc by nourishment status. As exploratory analyses, time to experiencing inpatient mortality is modelled on the same propensity score adjusted data sets.

#### Sensitivity analysis using an instrumental variable

As the CIN data are limited to the recorded variables, we use timing of admission [24,25] as an instrumental variable to examine the potential influence of any unmeasured variables in all the analysis of time to being discharged alive [24]. Multiple studies including Berkley (2004), Bell (2001), Freemantle (2015), Meacock (2016) and Aldridge (2016) have demonstrated that patients who were admitted during the weekend experienced worse outcomes compared to those admitted during the weekdays [26-30] – which may be an indication of poor quality of care and treatment during the weekend. This, in theory, implies that the type of treatment and care received depend on the day of admission – and which later determines the type of health outcome of the patient. See the supplementary material for the process followed in fitting the instrumental variable models.

## RESULTS

### Inclusion and exclusion

**Figure 1:**
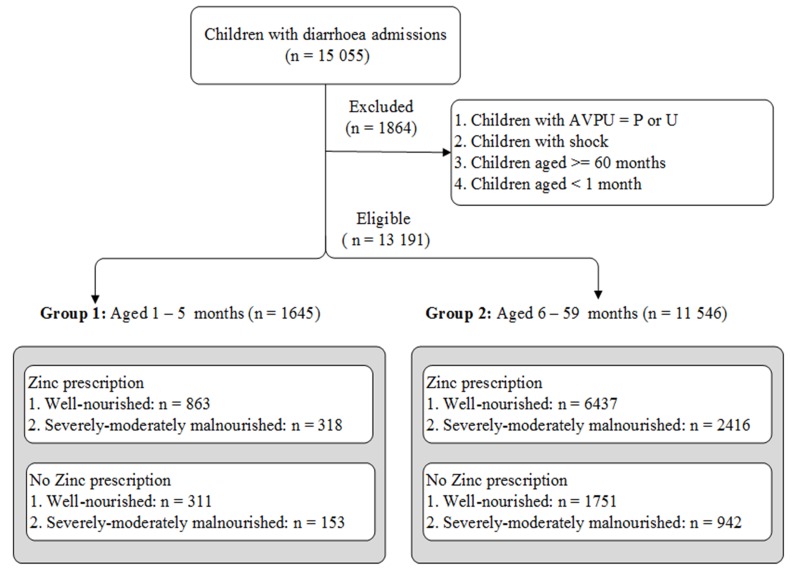
Eligibility criteria – shows the inclusion and exclusion criteria. The point of entry was a child with diarrhoea as one of the illnesses.

A total of 1645 and 11 546 children were eligible for analysis in groups 1 (aged 1-5 months) and 2 (aged 6-59 months) respectively. About 72% (1-5 months) and 77% (6-59 months) were prescribed zinc supplements (**Figure 1**). See detailed distribution of patients by clinical signs, cotreatments, comorbidities and hospital in **Table 1**.

### Outcome analysis

#### Exploring probability of being discharged alive in groups 1 and 2

**Figure 2:**
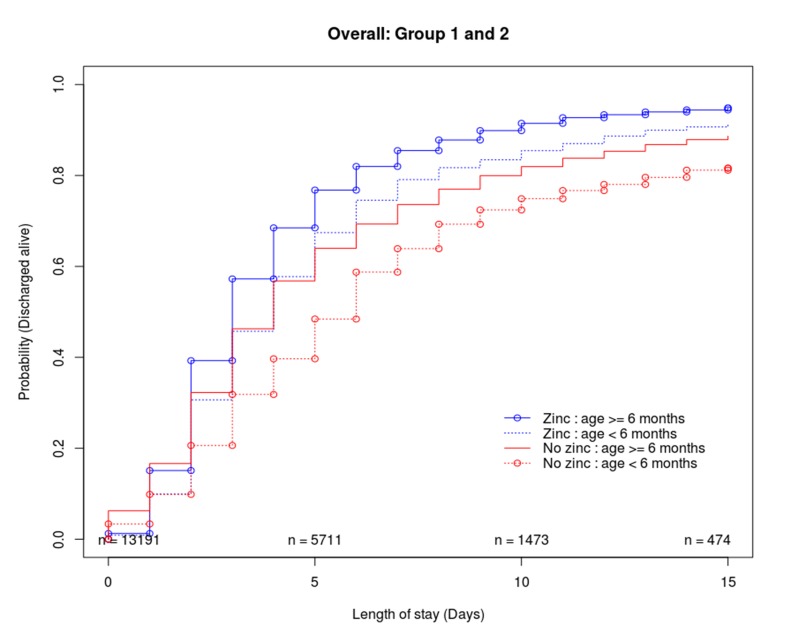
Probability of getting discharged alive (the cumulative incidence curves were estimated on data sets without propensity score adjustments). Those who were prescribed zinc were more likely to be discharged sooner.

Cumulative incidence functions were explored in the raw data sets to describe the probability of being discharged alive over time (**Figure 2**). Approximately 60% of the children were discharged by the fifth day of their stay in the hospital. Mortality was approximately 9% (zinc = 82/1181, non-zinc = 64/464) and 5% (zinc = 353/8853, non-zinc = 226/2693) in age groups 1 and 2 respectively. Approximately 2.6% (zinc = 25/1181, non-zinc = 18/464) and 1.8% (zinc = 119/8853, non-zinc = 84/2693) in groups 1 and 2 were censored as they either absconded, were referred to other hospitals and or had length of stay greater than 15 days. In both age groups (and without propensity score adjustment) children who were prescribed zinc were discharged sooner than those who were not prescribed zinc. However, this difference was not clear for those who were discharged within three days of hospitalisation.

#### Modelling time to discharge

**Figure 3:**
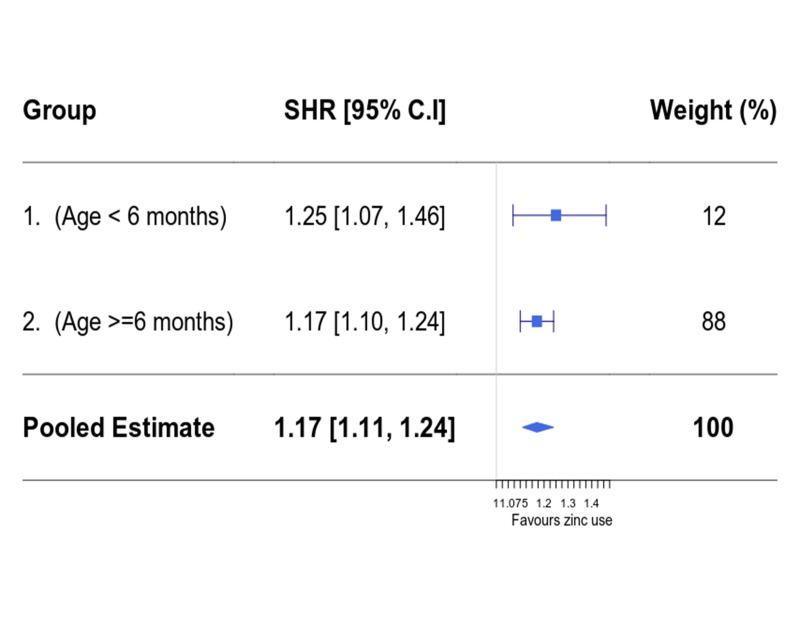
Estimated sub-distribution hazard ratios in age groups 1 and 2. Presents age specific subdistribution hazard ratio (SHR) as well as pooled estimates.

For group 1, the performance of optimal full matching was comparable to that of weighting, while weighting performed better than optimal full matching in group 2 (see **Table 1**, Figures S2 and S3 in **Online Supplementary Document**). Thus modelling time to discharge analyses for both age groups were based on propensity score weighted data sets. In propensity score weighted data sets, all the variables had ASMD <10% (apart from hospital variable in group 1) – which indicated the covariate imbalance between the zinc and non-zinc groups were minimised. The estimated sub-distribution hazard ratios (SHR) in multivariable PS weighted models (of being discharged alive in the zinc group vs the non-zinc group) were 1.25 (95% CI = 1.07, 1.46) and 1.17 (95% CI = 1.10, 1.24) in age groups 1 and 2 respectively. In a pooled analysis (across the two age groups), the overall effectiveness of zinc was 1.17 (95% CI = 1.11, 1.24). This can be interpreted as a child being prescribed zinc having on average a 17% higher chance of being discharged at any point in time than a child not prescribed zinc (**Figure 3**). Multivariable models without propensity score weighting produced somewhat higher effect estimates (SHR 1.33 (95% CI = 1.16, 1.53) and 1.30 (95% CI = 1.23, 1.38) for age groups 1 and 2, respectively) suggesting propensity score weighting may be adjusting for some potential confounding favouring zinc prescription.

#### Zinc effectiveness for well-nourished and under-nourished children

Approximately a third of children in both age groups were severely-moderately under-nourished (group 1 = 28.6% and group 2 = 29.1%). The additive interaction resulted in four sub-groups of children including those who were: (a) well-nourished with zinc prescribed; (b) well-nourished without zinc prescribed; (c) under-nourished with zinc prescribed and; (d) under-nourished without zinc prescribed. Patient characteristics were approximately similar across the four sub-groups in both age groups (see Tables S2 and S3 in **Online Supplementary Document**). The propensity score adjusted Scheike’s flexible model estimates (in both age groups) suggested that those who were prescribed zinc and were well-nourished were more likely to be discharged sooner, followed by those who were not prescribed zinc and were well-nourished, then those who were prescribed zinc but were under-nourished and lastly those who were not prescribed zinc but were under-nourished (**Table 2**). Results interpreted in a similar way were found using propensity score unadjusted data (see Table S4 in **Online Supplementary Document**).

**Table 2:** Propensity score adjusted SHR

	1-5 mo	6-59 mo
Zinc-undernourished*	0.82 (0.67, 1.01)	0.85 (0.79, 0.91)
No zinc-wellnourished*	0.88 (0.74, 1.04)	0.90 (0.84, 0.98)
No zinc-undernourished*	0.63 (0.49, 0.82)	0.64 (0.58, 0.70)
Zinc-wellnourished (reference group)*	-	-

SHR – subdistribution hazard ratio

*Zinc-undernourished – received zinc but were under-nourished, No zinc-wellnourished – were wellnourished but did not receive zinc, No zinc-undernourished – were undernourished but did not receive zinc, Zinc-wellnourished – received zinc and were well nourished.

#### Sensitivity analysis using an instrumental variable

The estimated SHRs (comparing zinc vs no zinc) using the instrumental variable for children aged 1-5 and 6-59 months were 1.24 (95% CI = 1.18, 1.30) and 1.31 (95% CI = 1.27, 1.35) respectively – and these were consistent with those obtained in **Figure 3**.

#### Modelling time to experiencing inpatient mortality (exploratory analysis)

**Figure 4:**
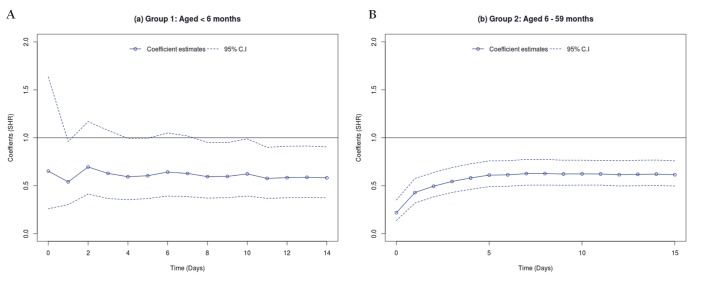
Estimated mortality time-varying subdistribution hazard ratio (SHR) with corresponding 95% confidence intervals (CI). The continuous lines (with points) show the estimated SHR coefficients by discharge time point and the non-continuous lines show the 95% CI. **Panel A.** Time varying mortality SHR in children aged 1-5 months. **Panel B.** Time varying mortality SHR in children aged 6-59 months.

Both Kolmogorov-Smirnov and Cramer Von Mises tests showed that the use of zinc had varying effects on mortality (across the discharge time points) both for children aged 1-5 and 6-59 months (see Table S5 in **Online Supplementary Document**). Thus, time specific estimates (with corresponding 95% C.I) were modelled using Scheike’s regression and the results presented in **Figure 4** (Panel A and Panel B). For children aged 1-5 months, zinc seemed to have no effect on mortality for those who stayed in the hospital for about a week. However, beyond one week, those who were prescribed zinc seemed to have significantly reduced risk of dying. While zinc use was associated with reduced mortality for children aged 6-59 months across all the discharge time points.

#### Analysis under missingness not at random

The Scheike’s model zinc treatment effectiveness estimates, under MNAR, were as presented in Figures S4, Panel A and B, in **Online Supplementary Document**. The pattern specific trends were in opposite directions although in group 2 the effect estimate was larger where there was most missingness. However, the pooled effects were consistent with those observed in **Figure 3**. This indicates that the earlier assumption of data missing at random was plausible.

## DISCUSSION

The results indicate that zinc may be beneficial in reducing time to discharge for children aged 1–59 months admitted with diarrhoea. This may be generalizable to children who have fever, normal pulse, are alert, vomiting, have capillary refill ≤3 minutes, those who do not require blood transfusion, and have no pallor, convulsions, thrush or oedema. An association with benefit is seen for both well-nourished and severely – moderately under-nourished children. This analysis supports and strengthens the current treatment policy used in Kenya which was implemented on the basis of limited evidence supporting the use of zinc in those aged less than 6 months and in well-nourished children. Two previous trials conducted in Africa (Ethiopia, n = 177, and Nigeria, n = 60) with children under 6 months [5,7] showed no effect of zinc in reducing diarrhoea duration. Our analyses offer the advantage of larger sample size but have the limitation of being based on a non-randomised study. Previous studies [7] have reported no serious adverse effects associated with the use of zinc apart from a risk of increased vomiting (within 10 minutes of administration). We were unable to examine differences in this possible adverse effect but if it occurs it does not seem to be increasing the length of stay for hospitalised children with diarrhoea. The findings therefore seem to contribute additional evidence on the likely value of zinc in support of its routine use for hospitalised children with diarrhoea in Kenya.

### Strengths and limitations

There are limitations whenever one uses routine, observational data sets. The WHO recommends two measures of malnutrition – m id-upper arm circumference and weight for height – but we were not able to use these as they are poorly documented in hospitals. Instead we used weight for age z score, associated with undernourishment, as a proxy. Data on specific covariables were also sometimes missing. We used multiple imputation to overcome this challenge assuming data were MAR. Methodologically, this assumption seemed plausible as estimates of the effects associated with zinc under assumptions of MAR and MNAR were approximately similar.

Comparative effectiveness analyses using observational data sets offer the potential advantage of evaluating “real world” effectiveness of treatments. In part, this is because children who may not qualify to be part of RCTs may be included in these observational studies. The use of propensity scores and multiple imputation provided a means to analytically handle the challenges of non-random allocation and missing data by creating samples of patients that are comparable in terms of *observed* signs, symptoms, co-treatments and comorbidities. Analyses of these samples provided biologically plausible results showing the benefits of zinc in reducing time to discharge for all children below five years. However, as analytic methods used in observational studies may not completely eliminate unobservable bias [31,32], we conducted sensitivity and exploratory analyses with the use of an instrumental variable and time to experiencing mortality respectively. The findings of instrumental variable analyses were consistent with those obtained with the propensity score methods. Also, our analysis demonstrated that zinc prescription was associated with reduced mortality – and this was consistent with the findings of a community trial in Bangladesh, which showed reduced chances of mortality by 50% [33]. These findings on mortality, however, are in contrast with those of RCTs included in the systematic reviews [7,34], which showed no effect of zinc on mortality. It may therefore be that residual bias is influencing our reported findings. It is plausible that zinc, an oral medication, is not prescribed for children who are thought to be more severely ill by clinicians based on their gut feeling rather than their recorded clinical features. Gut feeling has been identified as an important predictor of severe illness in some studies [35]. The possibility of this form of allocation bias is perhaps supported by the finding, in children 6-59 months, that zinc is associated with reduced mortality from the first day of admission as biologically zinc may not be expected to have such a rapid protective effect on this outcome.

## CONCLUSION

Our finding that zinc is associated with shorter time to discharge is consistent with meta-analyses of RCTs but extends this observation to less studied populations. RCTs have, however, not shown an advantageous effect of zinc on mortality in acute diarrhoea. The protective association of zinc against mortality we see does raise the possibility that there is residual (unobservable) bias in our observational analyses possibly due to clinicians allocating this treatment to less sick children. Despite this caveat, and as zinc is well tolerated and cheap, our results support the continued use of routine zinc supplementation in all children aged 1-59 months hospitalised with diarrhoea in Kenya and probably elsewhere in Africa.

## Additional material

Online Supplementary Document

## References

[1] International Vaccine Access Center. Pneumonia and Diarrhoea Progress Report 2014. Available: http://www.jhsph.edu/research/centers-and-institutes/ivac/resources/IVAC-2014-Pneumonia-Diarrhea-Progress-Report.pdf. Accessed: 11 December 2017.

[2] Ministry of Health. Kenya. Basic paediatric protocols 2016. Available: https://www.tropicalmedicine.ox.ac.uk/_asset/file/basic-paediatric-protocols-2016.pdf. Accessed: 13 December 2017.

[3] World Health Organisation. Hospital Care for Children 2013; Available: http://apps.who.int/iris/bitstream/10665/81170/1/9789241548373_eng.pdf. Accessed: 13 December 2017.

[4] Lazzerini M Ronfani L Oral zinc for treating diarrhoea in children. Cochrane Database Syst Rev. 2012;6:CD005436. 10.1002/14651858.CD005436.pub3 22696352

[5] Fischer Walker CL Bhutta ZA Bhandari N Teka T Shahid F Taneja S Zinc Supplementation for the Treatment of Diarrhea in Infants in Pakistan, India and Ethiopia. J Pediatr Gastroenterol Nutr. 2006;43:357-63. 10.1097/01.mpg.0000232018.40907.00 16954960

[6] Roy SK Tomkins AM Akramuzzaman SM Behrens RH Haider R Mahalanabis D Randomised controlled trial of zinc supplementation in malnourished Bangladeshi children with acute diarrhoea. Arch Dis Child. 1997;77:196-200. 10.1136/adc.77.3.196 9370894 PMC1717301

[7] Lazzerini M Wanzira H Oral zinc for treating diarrhoea in children. Cochrane Database Syst Rev. 2016;12:CD005436. 10.1002/14651858.CD005436.pub5 27996088 PMC5450879

[8] Sazawal S Black RE Bhan MK Bhandari N Sinha A Jalla S Zinc supplementation in young children with acute diarrhea in India. N Engl J Med. 1995;333:839-44. 10.1056/NEJM199509283331304 7651474

[9] Wessells KR Brown KH Estimating the global prevalence of zinc deficiency: results based on zinc availability in national food supplies and the prevalence of stunting. PLoS One. 2012;7:e50568. 10.1371/journal.pone.0050568 23209782 PMC3510072

[10] Patel A Mamtani M Dibley MJ Badhoniya N Kulkarni H Therapeutic value of zinc supplementation in acute and persistent diarrhea: a systematic review. PLoS One. 2010;5:e10386. 10.1371/journal.pone.0010386 20442848 PMC2860998

[11] Mwakyusa S Wamae A Wasunna A Were F Esamai F Ogutu B Implementation of a structured paediatric admission record for district hospitals in Kenya – results of a pilot study. BMC Int Health Hum Rights. 2006;6:9. 10.1186/1472-698X-6-9 16857044 PMC1555611

[12] Ayieko P Ntoburi S Wagai J Opondo C Opiyo N Migiro S A multifaceted intervention to implement guidelines and improve admission paediatric care in kenyan district hospitals: a cluster randomised trial. PLoS Med. 2011;8:e1001018. 10.1371/journal.pmed.1001018 21483712 PMC3071366

[13] World Health Organisation. Child growth standards 2011; Available: http://www.who.int/childgrowth/software/en/. Accessed 14 December 2017.

[14] Exuzides A Colby C An application of imputation techniques to improve data availability from electronic medical records. Value Health. 2010;13:A368 10.1016/S1098-3015(11)72483-4

[15] White IR Royston P Wood AM Multiple imputation using chained equations: Issues and guidance for practice. Stat Med. 2011;30:377-99. 10.1002/sim.4067 21225900

[16] Malla L Perera-Salazar R McFadden E Ogero M Stepniewska K English M Handling missing data in propensity score estimation in comparative effectiveness evaluations: a systematic review. J Comp Eff Res. 2018;7:271-9. 10.2217/cer-2017-0071 28980833 PMC6478118

[17] Stuart EA Matching methods for causal inference: a review and a look forward. Stat Sci. 2010;25:1-21. 10.1214/09-STS313 20871802 PMC2943670

[18] Austin PC An introduction to propensity score methods for reducing the effects of confounding in observational studies. Multivariate Behav Res. 2011;46:399-424. 10.1080/00273171.2011.568786 21818162 PMC3144483

[19] Austin PC Assessing balance in measured baseline covariates when using many-to-one matching on the propensity-score. Pharmacoepidemiol Drug Saf. 2008;17:1218-25. 10.1002/pds.1674 18972455

[20] Austin PC Balance diagnostics for comparing the distribution of baseline covariates between treatment groups in propensity-score matched samples. Stat Med. 2009;28:3083-107. 10.1002/sim.3697 19757444 PMC3472075

[21] Scheike TH Zhang MJ Analyzing competing risk data using the R timereg Package. J Stat Softw. 2011;38:i02. 10.18637/jss.v038.i02 22707920 PMC3375021

[22] Rubin DB An overview of multiple imputation. Proc J Am Stat Assoc. 1988;79-90. 10.1080/01944368808977158

[23] VanderWeele TJ Knol MJ A tutorial on interaction. Epidemiol Methods. 2014;3:33-72.

[24] Baiocchi M Cheng J Small DS Tutorial in biostatistics: instrumental variable methods for causal inference. Stat Med. 2014;33:2297-340. 10.1002/sim.6128 24599889 PMC4201653

[25] Malla L Perera-Salazar R McFadden E English M Comparative effectiveness of injectable penicillin versus a combination of penicillin and gentamicin in children with pneumonia characterised by indrawing in Kenya: a retrospective observational study. BMJ Open. 2017;7:e019478. 10.1136/bmjopen-2017-019478 29146662 PMC5695483

[26] Berkley JA Brent A Mwangi I English M Maitland K Marsh K Mortality among Kenyan children admitted to a rural district hospital on weekends as compared with weekdays. Pediatrics. 2004;114:1737-8. 10.1542/peds.2004-1263 15574646

[27] Bell CM Redelmeier DA Mortality among patients admitted to hospitals on weekends as compared with weekdays. N Engl J Med. 2001;345:663-8. 10.1056/NEJMsa003376 11547721

[28] Meacock R Anselmi L Kristensen S Doran T Sutton M Higher mortality rates amongst emergency patients admitted to hospital at weekends reflect a lower probability of admission. J Health Serv Res Policy. 2017;22:12-9. 10.1177/1355819616649630 27255144 PMC5482385

[29] Freemantle N Ray D McNulty D Rosser D Bennett S Keogh BE Increased mortality associated with weekend hospital admission: a case for expanded seven day services? BMJ. 2015;351:h4596. 10.1136/bmj.h4596 26342923

[30] Aldridge C Bion J Boyal A Chen Y Clancy M Evans T Weekend specialist intensity and admission mortality in acute hospital trusts in England: a cross-sectional study. Lancet. 2016;388:178-86. 10.1016/S0140-6736(16)30442-1 27178476 PMC4945602

[31] Cook TD Steiner PM case matching and the reduction of selection bias in quasi-experiments: The relative importance of pretest measures of outcome, of unreliable measurement, and of mode of data analysis. Psychol Methods. 2010;15:56-68. 10.1037/a0018536 20230103

[32] Suh HS Hay JW Johnson KA Doctor JN Comparative effectiveness of statin plus fibrate combination therapy and statin monotherapy in patients with type 2 diabetes: Use of propensity-score and instrumental variable methods to adjust for treatment-selection bias. Pharmacoepidemiol Drug Saf. 2012;21:470-84. 10.1002/pds.3261 22461130

[33] Larson CP Roy SK Khan AI Rahman AS Qadri F Zinc treatment to under-five children: applications to improve child survival and reduce burden of disease. J Health Popul Nutr. 2008;26:356-65. 18831230 10.3329/jhpn.v26i3.1901 PMC2740712

[34] Mayo-Wilson E Imdad A Junior J Dean S Bhutta ZA Preventive zinc supplementation for children, and the effect of additional iron: a systematic review and meta-analysis. BMJ. 2014;4:e004647. 24948745 10.1136/bmjopen-2013-004647 PMC4067863

[35] Van den Bruel A Thompson M Buntinx F Clinicians’ gut feeling about serious infections in children: observational study. BMJ Open. 2012;345:e6144. 23015034 10.1136/bmj.e6144 PMC3458229

