# Supplementary material for: Examining the effectiveness of zinc treatment in children admitted with diarrhoea in Kenya’s public hospitals: an observational comparative effectiveness study
Source: J Glob Health. 2019 Sep 17;9(2):020416. doi: 10.7189/jogh.09.020416 (PMC6748787; doi:10.7189/jogh.09.020416)
Supplement: Online Supplementary Document [file jogh-09-020416-s001.pdf]

Examining the effectiveness of Zinc treatment in children admitted with diarrhoea in Kenya's public hospitals: An observational study

The supplementary material is organised into the following subsections:

- Further information on Clinical Information Network
- Variables and their completeness (%)
- MNAR patterns
- Fitting of PS models
- Instrumental variable analysis
- Comparing performance of PS optimal full matching and weighting in groups 1 (1 – 5 months) and 2 (6 – 59 months) respectively
- Covariate balance across the four levels of additive interaction between Zinc prescription and nourishment status
- Treatment effect estimates by nourishment status
- Examining constant effects of Zinc in analysing time to experiencing inpatient mortality
- Pattern mixture modelling
- R packages used

a) Further Information on Clinical Information Network

Thirteen county referral hospitals plus one sub – county hospital were purposively selected with direction from Ministry of Health (MOH) and recruited into the CIN, which represents collaborative work between the MOH, the Kenya Medical Research Institute (KEMRI)/Wellcome Trust Research Programme, the Kenya Pediatric Association (KPA) and participating hospitals. These hospitals were recruited into the study at different times; four Nairobi sites in September 2013, six Western sites in October 2013 and four Central sites in February 2014 (Nairobi, Western and Central are three blocks in which the hospitals have been categorized).

The total possible variables collected per record are 382 and are grouped into; biodata, history, examination, investigations, admission diagnosis, treatment, supportive care, monitoring and discharge information tools. However, many variables are collected for only specific sub-groups (e.g. linked to diagnoses) or only if a child receives specific treatments. The total number of variables for which data are collected per patient is therefore well below this maximal figure of 382. The biodata tool contains basic patient's demographic information; history and examination has information on clinical signs and symptoms; investigations has laboratory test orders and results; and the discharge information tool contains discharge diagnoses, outcomes and follow up information.

This data collection system has been described in detail elsewhere(1). Feedback to hospitals as part of the CIN activities has helped improve the quality of clinical data (1). Details of hospital characteristics, their selection and their populations of patients are also provided elsewhere (2).

b) Variables and their completeness

Table S1 presents all the variables used for analysis. These data (for those aged 1 – 5 months and those aged 6 – 59 months together) had missing data of at most 23%.

Table S1: Percentage of missing data

**Variable**	**%**
Pulse^{d} (weak, normal)	10.2
AVPU^{d} (Alert, Verbal response, Pain response, Unresponsive)	4.5
Capillary refill^{d} (<=3 sec, >3 sec, intermediate)	17.0
Sunken eyes^{d} (Yes, No)	10.7
Skin pinch^{d} (1 – 2 sec, >2 sec, immediate)	8.8
Blood transfusion order^{d} (Yes, No)	0.3
Ability to drink^{d} (Yes, No)	8.8
Skin temperature^{d} (elbow, hand, shoulder)	21.9
Child sex^{d} (male, female)	0.8
Weight^{c} (Kg)	3.9
Pallor^{d} (none, mild/moderate, severe)	4.3
Temperature^{c} (degrees celcius)	10.3
Fever^{d} (Yes, No)	3.1
Convulsions^{d} (Yes, No)	5.6
Vomiting^{d} (Yes, No)	2.3
Hospital referral^{d} (Yes, No)	23.2
Length of illness (days)	2.3
Severe wasting^{d} (Yes, No)	21.6
Thrush^{d} (Yes, No)	13.1
Oedema^{d} (face, foot, knee, none)	6.7
Oral fluid^{d} (administered, not administered)	0.0
IV fluid^{d} (administered, not administered)	0.0
Wheeze^{d} (Present, absent)	4.9
Hospital^{d} (H 1 – 14)	0.0
Diarrhoea > 14 days^{d} (Yes,No)	14.4
HIV^{d} (positive, negative)	0.0
Pneumonia^{d} (positive, negative)	0.0
Malaria^{d} (positive, negative)	0.0
Meningitis^{d} (positive, negative)	0.0

^{d} represent discrete variable while ^{c} represents continuous variable; major co – morbidities included HIV, pneumonia, malaria and meningitis; fluid treatments included – IV and oral fluid. Missingness in outcome (alive, dead, absconded, referred) was 1.3%, age (months) was 0.4%, and in length of stay 0.9%.

After assessing completeness of the variables, 10 datasets were multiply imputed using chained equations (though no higher order forms of continuous variables or interactions are included). Thereafter, average densities of imputed values versus those observed were examined for the following continuous variables: length of illness,

age, weight and temperature (Figure S1). The densities for observed and imputed values were overlapping showing the plausibility of the imputed values and that the imputation models had worked correctly.

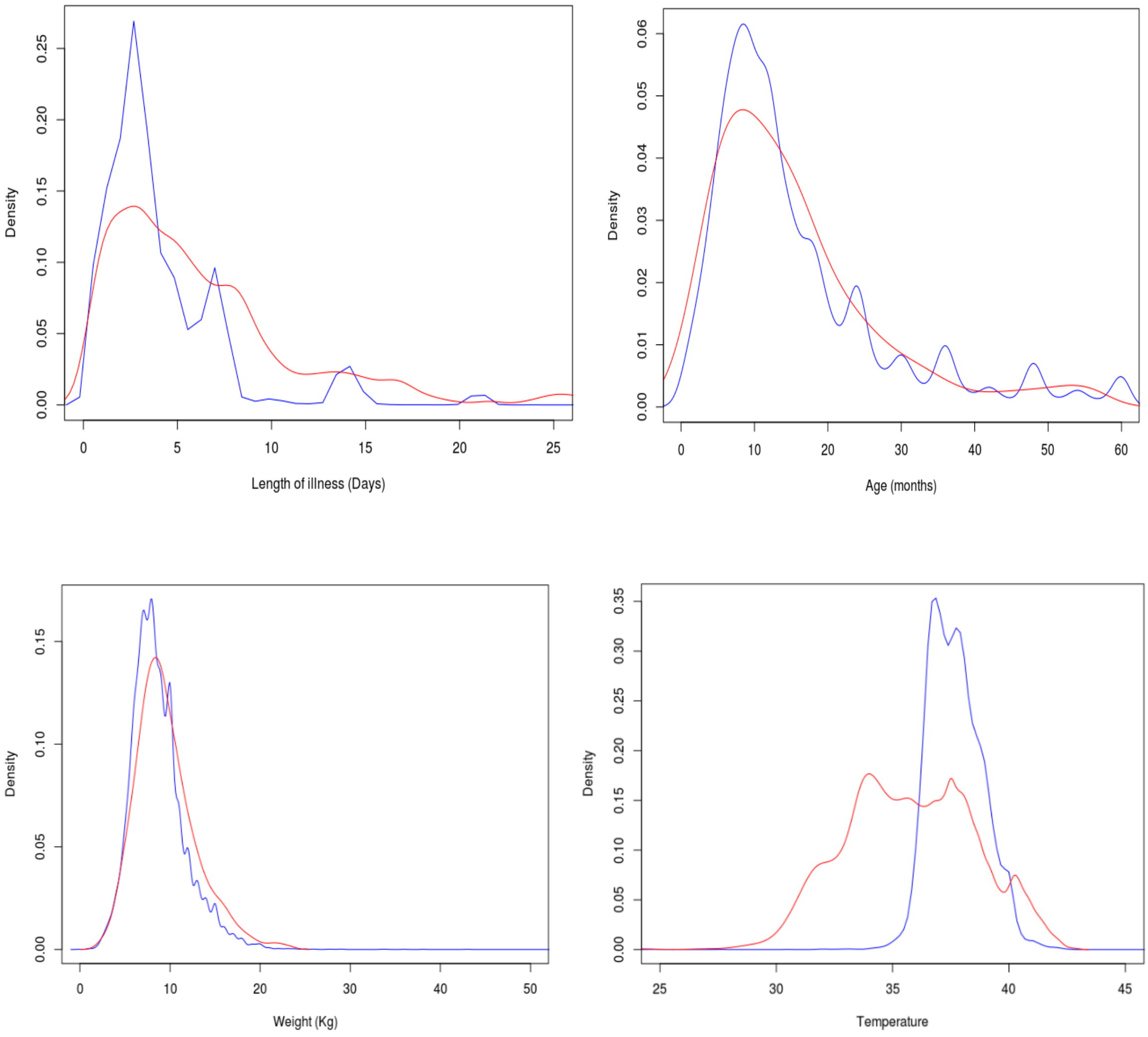

Figure S1: Distribution of observed versus average imputed values (continuous variables, blue line – observed data, red line – imputed data)

c) MNAR patterns

The use of patterns of missingness within the data supposes that there are unmeasured factors that create much more missingness in some medical records than in others so there is an underlying non – random factor. Thus conducting multiple imputation and subsequent analyses on data subsets that display different patterns of missingness may provide an indication (if results vary) that assumption of missingness at random may not be feasible(3). Molenberghs (2004) (3) indicate that the use of pattern mixture models may play a major role in missing data sensitivity analysis. In this analysis, pattern mixture models involve formulation of different missing data patterns – and outcome analyses are then conducted for each pattern and pooled. This analysis involves missing data in more than one covariate and therefore patterns are formulated by grouping of patients according to the number of variables for which they have missing data. This form of grouping of patients for missingness not at random was implemented in Gathara (2017)(4). In particular, three patterns are formulated in which pattern 1 consists of patients with missing data in 0 – 3 variables, pattern 2 consists of patients with 4 variables with missing data, and pattern 3 consists of patients with more than four variables with missing data. See sub – section k for results of the pattern mixture modelling.

d) Fitting of propensity score models

Logistic regression model with a logit link function was used to estimate the propensity scores. All the variables listed in Table S1 were used as independent variables in the PS model with the Zinc prescription being the dependent variable (the variable types were maintained in the analysis). The estimated propensity scores were then used to conduct PS full-matching and weighting. Full matching retained all the patients in the analysis while forming subsets of Zinc and non-Zinc patients using the optimal algorithm. Also we conducted weighting by odds such that those prescribed Zinc were assigned a weight of 1 and those in the non-Zinc assigned weights of PS/(1 – PS) (5). Weighting by odds results into stable weights thus does not require stabilisation.

e) Instrumental variable analysis:

A valid IV should satisfy the following three conditions: (i) it should be usable as a variable for randomly and effectively assigning patients into alternative groups (and this is to ensure that the IV is not influenced by any unobserved variables so it helps mimic the case of a randomised controlled trial); (ii) relevance – as the choice of IV should be logical and have a direct effect on treatment received and; (iii) it should not be directly associated with the outcome but only through the treatment (6). According to Baiocchi (2014), assumption one may partly be verified by examining covariate distribution across the levels of an IV variable and assumption two could be examined using likelihood ratio tests. And according to Klungel (2015), the third assumption may not be directly verifiable (7) but could be theoretically justified.

To assess whether admission timing forms a natural and random experiment the distributions of covariates were examined across the levels of the instrumental variable (grouped as weekend/weekday) for children aged 1 – 5 and 6 – 59 months separately. The distribution of each of the patient characteristics was approximately similar with the majority of variables having absolute standardised mean differences of <= 0.1 between groups. Likelihood ratio tests also showed that admission timing was significantly associated with Zinc prescription in both age groups.

To model using instrumental variables in a survival context, Tchetgen (2015) (8) suggested the use of control function where modelling happens in two steps. Step 1 involves the estimation of residuals. The residuals are estimated from a model where treatment membership is modelled as a function of the IV and covariates influencing treatment assignment (all the variables in Table S1 plus the admission timing variable were used in estimating the residuals). Step 2 involves the use of these residuals as predictors in a second stage survival model – and in this case we used the Scheike's flexible competing risk model.

The estimated SHRs (comparing Zinc versus no Zinc) using this IV for children aged 1 – 5 and 6 – 59 months were 1.24 [1.18, 1.30] and 1.31 [1.27, 1.35] respectively. These results are similar to those reported in figure 3 of the main manuscript. Consistent results were also obtained in IV analysis of Zinc effectiveness by nourishment status.

f) **Comparing performance of PS optimal full matching and weighting in groups 1 (1 – 5 months) and 2 (6 – 59 months) respectively**

We compared the ability of two PS approaches to reduce possible bias – optimal full matching and weighting (5, 9). Both are aimed at creating groups of patients that are comparable in terms of the distribution of observed signs and symptoms (though they may result in somewhat different groups being compared). In order to select the optimum PS implementation method, standardised mean differences were used as diagnostic checks for covariate balance and overlap (10, 11) between Zinc and non-Zinc groups. Even though both the PS methods would retain all patients in the analysis, the method that resulted in the minimum average absolute standardised mean differences for the majority of the variables was considered the most appropriate (5). For group 1 (1 – 5 months), the performance of optimal full matching was comparable to that of weighting, while weighting performed better than optimal full matching in group 2 (6 – 59 months) (see Figures S1 and S2). Thus outcome analyses for groups 1 and 2 were based on PS weighted datasets.

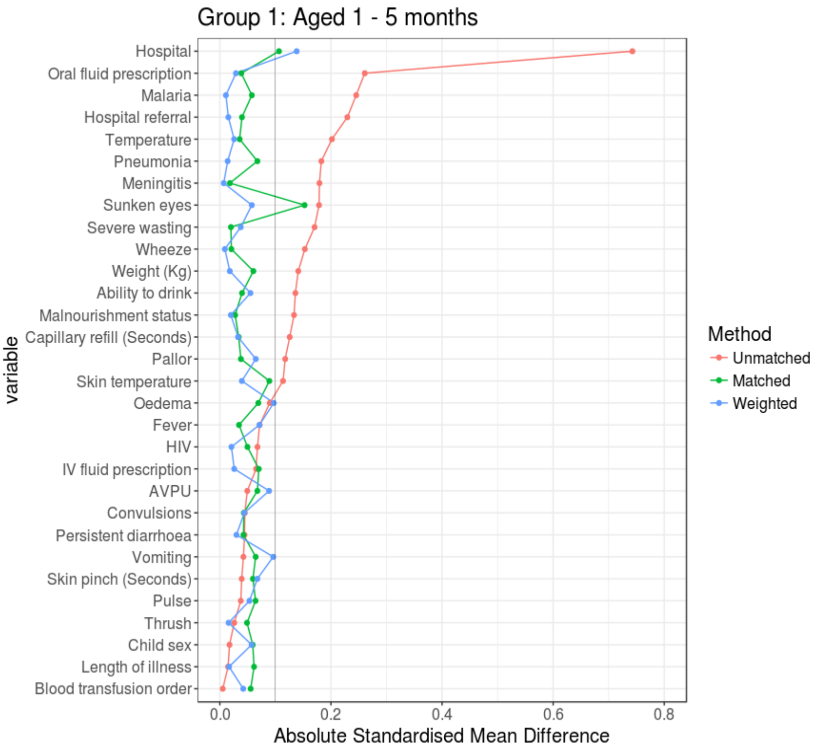

Figure S2: Comparing performance of the two PS implementation methods in group 1 (1 – 5 months). The y – axis contains all the variables used in the PS models. While x – axis shows absolute standardised mean difference (ASMD) which is a measure of covariate balance between Zinc and non-Zinc groups. An ASMD value of <= 0.1 indicates the method has performed well in creating comparable groups.

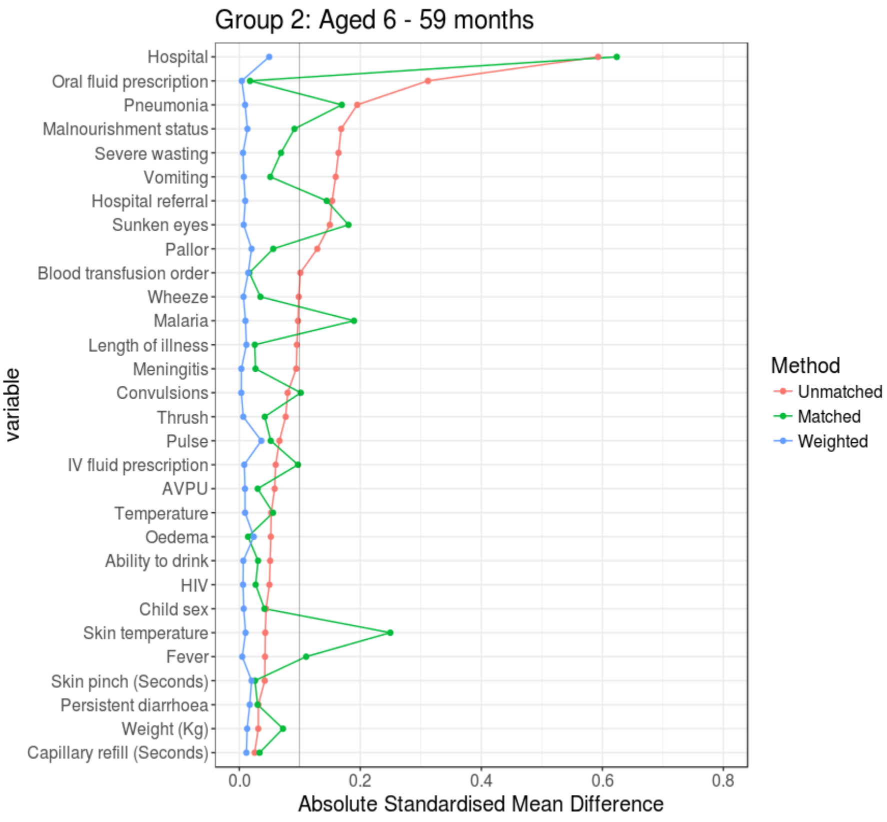

Figure S3: Comparing performance of the two PS implementation methods in group 2 (6 – 59 months)

g) Covariate balance across the four levels of additive interaction between Zinc prescription and nourishment status

To ensure the four subgroups had comparable patient characteristics, we followed the step by step approach suggested by Spreeuwenberg (2010) (12). This involved: (i) Fitting PS models using multinomial logistic regression with a probit link function to obtain four propensity scores per patient (which should add to one); (ii) Examining covariate balance across the four subgroups through the use of significance testing and only p – values reported. Based on the p – values, the distribution of covariates seem to be similar across the four sub – groups. See Tables S2 and S3.

Table S2: Patient distributions across the four subgroups and p – values for testing differences before and after PS adjustments (1 – 5 months)

	Zinc-wellnourished	Zinc-under-nourished	No zinc-wellnourished	No zinc-under-nourished	Before multiple PS correction (p - value)	After multiple PS correction (p - value)
**Pulse**						
Normal	95	91	96	92	0.050	0.998
Weak	5	9	4	8		
**AVPU**						
Alert	98	96	97	97	0.307	1.000
Verbal response	2	4	3	3		
**Capillary refill**						
<= 3 Sec	95	94	97	94	0.249	1.000
> 3 Sec	1	1	1	3		
Indeterminate	4	5	2	3		
**Sunken eyes**						
No	73	63	82	69	0.000	0.989
Yes	27	37	18	31		
**Skin pinch**						
1 -2 secs	21	26	20	29	0.000	1.000
Immediate	70	58	74	53		
more than or equal to 2secs	9	16	6	18		
**Blood transfusion order**						
No	98	95	98	96	0.090	0.999
Yes	2	5	2	4		
**Ability to drink**						
No	15	19	18	27	0.008	1.000
Yes	85	81	82	73		
**Skin temperature**						
Elbow	3	4	3	5	0.230	1.000
Hand	96	94	94	92		
Shoulder	1	2	3	3		
**Child sex**						
Female	46	42	49	39	0.142	0.999
Male	54	58	51	61		
**Pallor**						
mild/moderate	7	15	9	17	0.001	1.000
None	89	82	89	81		
Severe	3	3	2	2		
**Fever**						
No	19	20	20	27	0.173	1.000
Yes	81	80	80	73		
**Convulsions**						
No	92	93	89	95	0.053	0.999
Yes	8	7	11	5		
**Vomiting**						
No	39	42	39	44	0.648	1.000
Yes	61	58	61	56		
**Hospital referral**						
No	83	81	75	69	0.000	1.000
Yes	17	19	25	31		
**Severe wasting**						
No	97	83	95	80	0.000	0.909
Yes	3	17	5	20		
**Thrush**						
No	97	95	96	94	0.275	1.000
Yes	3	5	4	6		
**Oedema**						
Face	0	0	0	0	0.057	0.116
Foot	1	0	2	0		
Knee	0	0	0	0		
None	99	100	98	100		
**Oral fluid treatment**						
No	12	21	20	31	0.000	0.997
Yes	88	79	80	69		
**IV fluid treatment**						
No	71	57	68	59	0.000	0.996

Yes	29	43	32	41		
**Wheeze**						
No	97	95	90	97	0.001	0.999
Yes	3	5	10	3		
**History of pesistent diarrhoea**						
No	97	96	99	97	0.135	0.999
Yes	3	4	1	3		
**HIV**						
hiv+	1	3	1	5	0.001	0.995
hiv-	99	97	99	95		
**Pneumonia**						
pneum+	49	50	57	61	0.010	1.000
pneum-	51	50	43	39		
**Malaria**						
malaria+	25	16	17	7	0.000	0.998
malaria-	75	84	83	93		
**Meningitis**						
meningitis+	4	5	9	8	0.007	1.000
meningitis-	96	95	91	92		
**Continuous variables: mean (sd)**						
Weight	6 (2)	4 (1)	6 (2)	4 (1)	0.000	0.644
Temperature	38 (1)	38 (1)	38 (1)	38 (1)	0.002	0.999
Length of illness	5 (5)	7 (12)	5 (5)	7 (12)	0.000	0.998

Table S3: Patient distributions across the four subgroups and p – values for testing differences before and after PS adjustments (6 – 59 months)

	**Zinc-wellnourished**	**Zinc-under-nourished**	**No zinc-wellnourished**	**No zinc-under-nourished**	**Before multiple PS correction (p - value)**	**After multiple PS correction (p - value)**
**Pulse**						
Normal	95	93	94	91	0.000	0.962
Weak	5	7	6	9		
**AVPU**						
Alert	98	97	97	96	0.008	0.999
Verbal response	2	3	3	4		
**Capillary refill**						
<= 3 Sec	95	95	95	95	0.503	1.000
> 3 Sec	1	1	1	1		
Indeterminate	4	4	4	4		
**Sunken eyes**						
No	67	61	75	67	0.000	0.985
Yes	33	39	25	33		
**Skin pinch**						
1 -2 secs	23	23	22	24	0.000	0.982
Immediate	71	64	72	60		
more than or equal to 2secs	7	13	7	16		
**Blood transfusion order**						
No	98	97	96	95	0.000	0.999
Yes	2	3	4	5		
**Ability to drink**						
No	14	17	18	18	0.000	0.986
Yes	86	83	82	82		
**Skin temperature**						
Elbow	3	5	4	6	0.000	1.000
Hand	95	93	94	92		
Shoulder	2	2	2	3		
**Child sex**						
Female	46	40	48	43	0.000	0.987
Male	54	60	52	57		
**Pallor**						
mild/moderate	8	12	9	16	0.000	0.999
None	90	85	87	80		
Severe	2	3	4	4		
**Fever**						

No	24	22	20	24	0.007	0.990
Yes	76	78	80	76		
**Convulsions**						
No	88	91	85	89	0.000	0.999
Yes	12	9	15	11		
**Vomiting**						
No	19	20	24	29	0.000	1.000
Yes	81	80	76	71		
**Hospital referral**						
No	84	83	80	75	0.000	0.999
Yes	16	17	20	25		
**Severe wasting**						
No	98	85	97	77	0.000	0.060
Yes	2	15	3	23		
**Thrush**						
No	97	95	96	94	0.000	0.981
Yes	3	5	4	6		
**Oedema**						
Face	0	0	1	0	0.000	1.000
Foot	2	0	3	0		
Knee	0	0	0	0		
None	98	100	96	100		
**Oral fluid treatment**						
No	11	15	25	26	0.000	0.877
Yes	89	85	75	74		
**IV fluid treatment**						
No	69	62	65	62	0.000	0.939
Yes	31	38	35	38		
**Wheeze**						
No	98	97	96	96	0.000	0.999
Yes	2	3	4	4		
**History of pesistent diarrhoea**						
No	97	96	97	96	0.002	0.993
Yes	3	4	3	4		
**HIV**						
hiv+	1	3	1	3	0.000	0.763
hiv-	99	97	99	97		
**Pneumonia**						
pneum+	28	36	37	43	0.000	0.909
pneum-	72	64	63	57		
**Malaria**						
malaria+	31	18	27	17	0.000	0.869
malaria-	69	82	73	83		
**Meningitis**						
meningitis+	4	5	7	6	0.000	0.992
meningitis-	96	95	93	94		
**Continuous variables: mean (sd)**						
Weight	10 (3)	7 (2)	10 (3)	7 (2)	0.000	0.000
Temperature	38 (2)	38 (1)	38 (1)	38 (1)	0.000	0.992
Length of illness	4 (7)	6 (11)	5 (11)	7 (13)	0.000	0.943

h) Treatment effect estimates by nourishment status

An additive interaction was used in modelling the use of zinc by nourishment status (13). As such, we derived a variable with four levels representing those who were: (i) prescribed Zinc and were well-nourished; (ii) prescribed Zinc and were undernourished; (iii) not prescribed Zinc and were well-nourished and; (iv) not prescribed Zinc and were undernourished. The reference group used in the analyses, was well-nourished children who were prescribed Zinc as they were more likely to be discharged sooner than the other three subgroups. To ensure the four subgroups had comparable patient characteristics, we followed the step by step approach suggested by Spreeuwenberg (2010) (12). The distribution overlap and covariate balance were satisfactory as demonstrated in Tables S2 and S3. The treatment effect results obtained are presented in table 1 of the main manuscript. While results obtained with PS unweighted data are presented in Table S4 below.

Table S4: Treatment effect estimates (SHR) by nourishment status (unweighted data)

	**1 – 5 months**	**6 – 59 months**
Zinc-undernourished	0.52 [0.44, 0.61]	0.57 [0.54, 0.60]
No Zinc-wellnourished	0.74 [0.63, 0.87]	0.83 [0.77, 0.90]
No Zinc-undernourished	0.41 [0.33, 0.51]	0.41 [0.38, 0.45]
Zinc-wellnourished (reference group)	-	-

i) Examining constant effects of Zinc in analysing time to experiencing inpatient mortality

Using both Kolmogorov-Smirnov and Cramer von Mises tests, it was indicated that the effect of Zinc on mortality was not constant across the discharge time points (for both age groups). See Table S5.

Table S5: P – values for PS adjusted models

	**Group 1 (1 – 5 months)**		**Group 2 (6 – 59 months)**	
	**Kolmogorov-Smirnov**	**Cramer Von Mises**	**Kolmogorov-Smirnov**	**Cramer Von Mises**
Zinc prescription (Yes)	**0.01**	**0.01**	**0.00**	**0.00**

j) Pattern mixture modelling

The sample sizes per pattern were as presented in Table S6. Pattern 1 in both groups had the largest sample size, followed by pattern 2 and lastly pattern 3.

Table S6: Sample size per pattern

Pattern	Group 1	Group 2
1	758	5700
2	346	2140
3	541	3706
Total	1645	11546

PS weighting was conducted (to be consistent with the primary analysis) and this minimised covariate imbalance across the three patterns in group 2 and all the variables had ASMD < =10%. However, about a third and half of the variables in patterns 1 and 2 respectively in group 1 had ASMD > 10%, and six variables had ASMD > 10% in pattern 3 of group 1. The Scheike's model Zinc treatment effectiveness estimates were as presented in Figures S4 a and b. The pattern specific trends were in opposite directions although in group 2 the effect estimate was larger where there was most missingness. However, the pooled effects were consistent with those observed in Figure 3 of the main manuscript. This indicates that the earlier assumption of data missing at random was plausible.

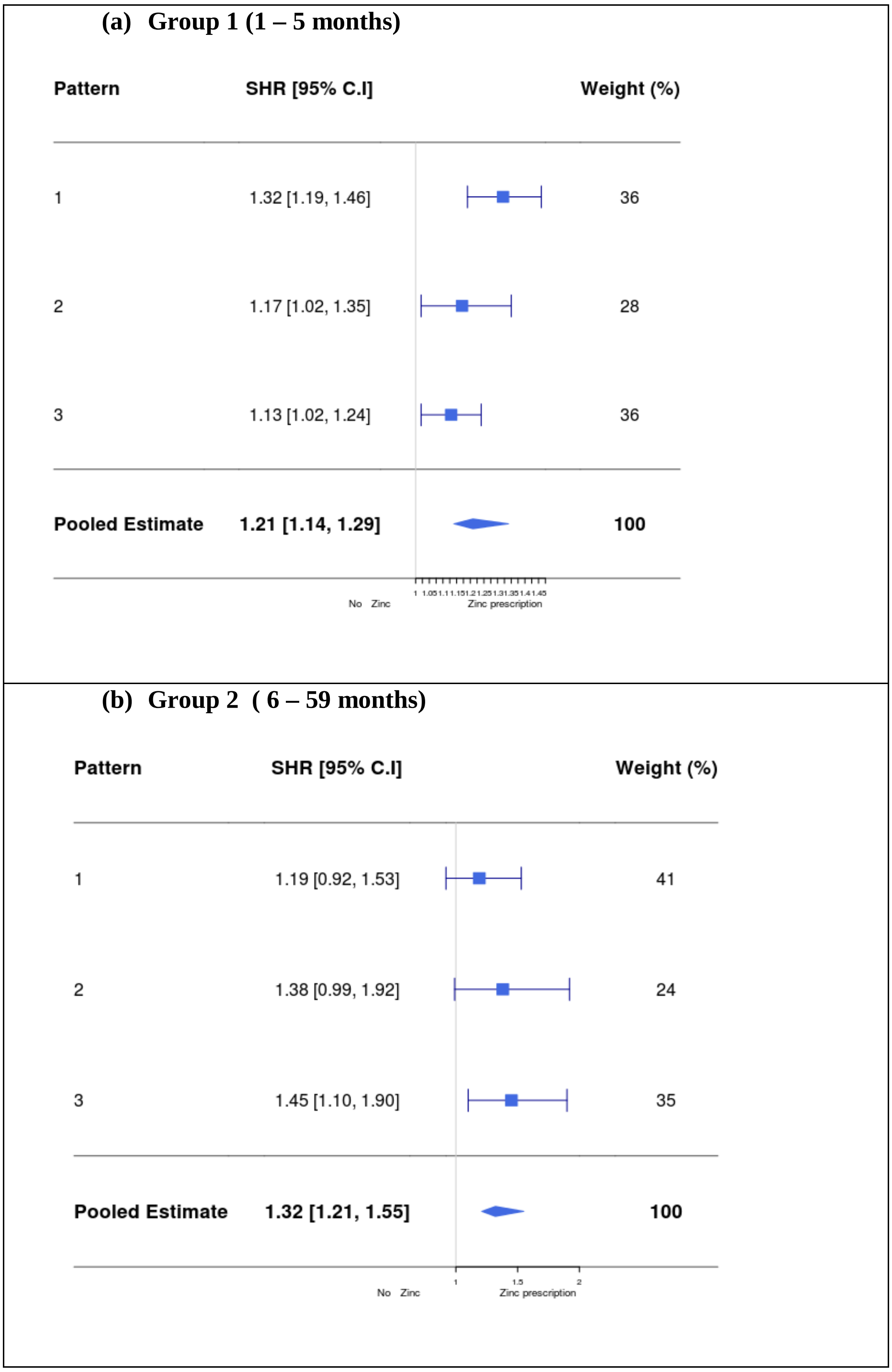

Figures S4 a and b: The estimated SHR per pattern in groups 1 and 2. Interpretation is based on the pooled effects rather than pattern specific.

k) R packages

We used the following R packages for analysis:

- timereg – for fitting the competing risk regression
- mice – for multiple imputation
- MatchIt – for conducting optimal full matching

References

1. Tuti T, Bitok M, Malla L, Paton C, Muinga N, Gathara D, et al. Improving documentation of clinical care within a clinical information network: an essential initial step in efforts to understand and improve care in Kenyan hospitals. BMJ Global Health. 2016;1(1):e000028.

2. Ayieko P, Ogero M, Makone B, Julius T, Mbevi G, Nyachiro W, et al. Characteristics of admissions and variations in the use of basic investigations, treatments and outcomes in Kenyan hospitals within a new Clinical Information Network

. Archives of Disease in Childhood. 2015;101(3):223-9.

3. Molenberghs G, Thijs H, Jansen I, Beunckens C, Kenward MG, Mallinckrodt C, et al. Analysing incomplete longitudinal clinical trial data. Biostatistics. 2004;5(3):445-64.

4. Gathara D, Malla L, Ayieko P, Karuri S, Allen E, Irimu G, et al. Variation in and risk factors for paediatric inpatient all-cause mortality in a low income setting: Data from an emerging clinical information network. . 2017;17(1):99.

5. Stuart EA. Matching methods for causal inference:A review and a look forward. Statist. Sci. 2010;25(1):1-21.

6. Baiocchi M, Cheng J, Small DS. Tutorial in Biostatistics: Instrumental Variable Methods for Causal Inference. Statistics in Medicine. 2014;33(13):2297-340.

7. Klungel OH, Jamal U.M., De Boer A., Belitser SV, Groenwold RH, Roes KC. Instrumental Variable Analysis in Epidemiologic Studies: An Overview of the Estimation Methods. Pharm Anal Acta. 2015;6:353.

8. TchetgenTchetgen EJ, Walter S, Vansteelandt S, Martinussen T, Glymour M. Instrumental variable estimation in a survival context. *26*(3), 402–410. Epidemiology (Cambridge, Mass.). 2015;26(3):402-10.

9. Austin PC. An Introduction to Propensity Score Methods for Reducing the Effects of Confounding in Observational Studies. Multivariate Behavioral Research. 2011;46(3):399-424.

10. Austin PC. Assessing balance in measured baseline covariates when using many-to-one matching on the propensity-score. Pharmacoepidemiol Drug Saf. 2008 2008;17(12):1218-25.

11. Austin PC. Balance diagnostics for comparing the distribution of baseline covariates between treatment groups in propensity-score matched samples. Stat Med. 2009 10 Nov 2009;28(25):3083-107.

12. Spreeuwenberg MD, Bartak A, Croon MA, Hagenaars JA, Busschbach JJ, Andrea H, et al. The multiple propensity score as control for bias in the comparison of more than two treatment arms: An introduction from a case study in mental health. Med. Care. 2010;48:166-74.

13. VanderWeele TJ, Knol MJ. A Tutorial on Interaction. Epidemiol. Methods. 2014;3(1):33-72.
